# Neutrophil-to-lymphocyte ratio in relation to the risk of all-cause mortality and cardiovascular events in patients with chronic kidney disease: a systematic review and meta-analysis

**DOI:** 10.1080/0886022X.2020.1832521

**Published:** 2020-10-20

**Authors:** Wen-Man Zhao, Shu-Man Tao, Gui-Ling Liu

**Affiliations:** Department of Nephrology, The Second Hospital of Anhui Medical University, Hefei, China

**Keywords:** All-cause mortality, cardiovascular events, meta-analysis, neutrophil-to-lymphocyte ratio

## Abstract

**Aim:**

To systematically evaluate the relationship between the neutrophil-to-lymphocyte ratio (NLR) and the risk of all-cause mortality or cardiovascular events in patients with chronic kidney disease (CKD).

**Methods:**

PubMed, Embase, and Web of Science databases were searched for cohort studies that were published since the databases were launched, until 1 April 2020. We selected papers according to specific inclusion and exclusion criteria, extracted data, and evaluated the quality of the citations. Data from eligible studies were used to calculate the combined hazard ratios (HRs) and 95% confidence intervals (CI).

**Results:**

The search identified 1048 potentially eligible records, and 10 studies (*n* = 1442) were selected. Eight studies reported all-cause mortality, and two studies reported cardiovascular events. The combined HR of all-cause mortality was 1.45 (95% CI 1.20–1.75) and the HR of cardiovascular events was 1.52 (95% CI 1.33–1.72) when NLR was considered as a categorical variable. Similarly, the association between NLR and all-cause mortality was confirmed (HR 1.35; 95% CI 1.23–1.48) when NLR was used as a continuous variable.

**Conclusion:**

NLR is a predictor of all-cause mortality and cardiovascular events in patients with chronic kidney disease.

## 1. Introduction

Chronic inflammation is exceedingly common in patients with chronic kidney disease (CKD) and is closely associated with all-cause mortality and poor prognoses of cardiovascular disorders [1–3]. Inflammation is an important initiating factor in the development of tubulointerstitial fibrosis, which eventually leads to disease progression [4].

Several factors contribute to the development of inflammation in patients with CKD, including an increased production of pro-inflammatory cytokines, chronic infections, oxidative stress, and acidosis. Several markers of inflammation, such as C-reactive protein (CRP) [5] and interleukin-6 [6], have been identified as reliable biomarkers and independent predictors of systemic inflammation showing prognostic value in patients with CKD. For example, a large multicenter international study of hemodialysis patients showed a positive correlation between CRP levels and mortality [7]. These mediators stimulate the glomerular endothelial and mesangial cells, subsequently damaging the glomerular basement membrane and increasing the production, and decreasing the degradation, of the endothelial extracellular matrix that can eventually lead to glomerular hypertension, renal tubule interstitial fibrosis, and kidney scarring. Since chronic inflammation plays a major role in the development and progression of CKD, it is important to assess and alleviate the extent of chronic inflammation to reduce the progression of kidney dysfunction [8].

The neutrophil-to-lymphocyte ratio (NLR), which can be obtained from routine blood tests, has attracted attention because of its wide availability and the low cost of the tests; it has recently emerged as a prognostic marker in various chronic diseases [9]. Studies have demonstrated that NLR is associated with the clinical outcome in patients with CKD; however, the conclusions of these studies are inconsistent [10–13]. Previous meta-analyses showed that NLR is associated with an increased risk of all-cause mortality in patients undergoing angiography or cardiovascular reconstruction or suffering from cerebral hemorrhage [14,15]. However, there was no meta-analysis of NLR and the risk of death in patients with CKD. Cardiovascular and cerebrovascular events are the common causes of death in patients with CKD [16]. Therefore, it is reasonable to hypothesize that high NLR values may be related to the prognosis of patients with CKD. In this study, we conducted a meta-analysis of eligible cohort studies to assess the association between NLR and the prognosis of patients with CKD.

## 2. Methods

### 2.1. Search strategy and eligibility criteria

A comprehensive search was conducted using PubMed, Embase, and Web of Science databases, covering reports published since the launch of the databases until April 1st, 2020. The search terms used were as follows: ‘the neutrophil to lymphocyte ratio’, ‘the ratio of neutrophil to lymphocyte’, ‘NLR’, ‘neutrophil/lymphocyte ratio’, ‘chronic kidney disease’, ‘chronic kidney failure’, ‘chronic renal failure’, ‘end-stage renal disease’, ‘hemodialysis’, ‘peritoneal dialysis’, or ‘uremia’. The search keywords were input as both medical subject headings (MeSH) and text words, without being restricted by ethnicity or geographic area. References cited in the included papers were examined for eligible articles. Retrieved papers were first screened independently by two unblind investigators at the title and abstract level, with disagreements resolved by a third reviewer. This review has been registered in the database INPLASY (DOI: 10.37766/inplasy2020.6.0112). All analyses were based on previous published studies, thus no ethical approval and patient consent are required.

Inclusion criteria were as follows: (all of the criteria were mandatory): (1) studies were prospective or retrospective cohort studies; (2) the participants were patients with CKD, including end-stage renal disease (ESRD) and dialysis population, and there was no restriction on gender, race, age, and occupation; (3) the outcome included any cause of death or the occurrence of major cardiovascular events; and (4) the study reported a baseline NLR and multiple adjusted hazard ratio (HR) with 95% CI for NLR. Exclusion criteria were (a single criterion resulted in exclusion): (1) research without available data, or (2) young patients (<18 years old) or animals.

### 2.2. Data extraction and quality evaluation

We extracted the following information from every paper: author name, year of publication, gender and age of target participants, location of patients, sample size, NLR grouping, outcome assessment, the follow-up period, and adjusted HR with 95% CI. The Newcastle-Ottawa Scale (NOS) was used to assess the quality of citations in this study by two investigators independently (W.Z. and S.T.). Studies with NOS scores of more than 6 were rated as high quality [17]. In case of disagreement, the issue was resolved by a third reviewer (G.L.).

The Cohen’s kappa statistic was used to assess the preliminary agreement between the two initial NOS scores. The kappa coefficient was considered almost perfect at 0.81–1.00, or substantial agreement at 0.61–0.80, moderate agreement at 0.41–0.60, fair agreement at 0.21–0.40, slight agreement at 0.01–0.20, and less than chance at <0. The quality of evidence across studies and the risk of bias for individual studies were independently assessed by two study authors.

### 2.3. Definition of outcomes

The primary outcomes were cardiovascular diseases (CVD) or death after follow-up. CVD events are defined as cardiovascular, cerebrovascular, or peripheral vascular diseases, including stroke, death, myocardial infarction, transient ischemic attacks, and peripheral vascular accidents. The secondary outcomes were kidney transplantation, loss of follow-up, and end of the study.

### 2.4. Statistical analyses

Data analyses were performed using STATA 15.1 (StataCorp). The *I^2^* statistic was used to identify heterogeneity. If *I*^2^ ≥ 50% or *p* < 0.05 was observed, a random effect model was used; otherwise, a fixed effect model was applied. Additionally, low, moderate, and high levels were nominally applied to define *I*^2^ values as 25%, 50%, and 75%, respectively [18]. Subgroup analyses were conducted to explore the sources of heterogeneity. Publication bias was inspected using a funnel plot. An egger’s test with *p* < 0.10 was considered as evidence of bias [19,20]. Sensitivity analysis was utilized to estimate the influence of a single study on the overall risk investigation and was conducted by sequentially omitting one study. Finally, a *p* < 0.05 was considered to be a significant difference.

## 3. Results

### 3.1. Literature search and study characteristics

**Figure 1.**
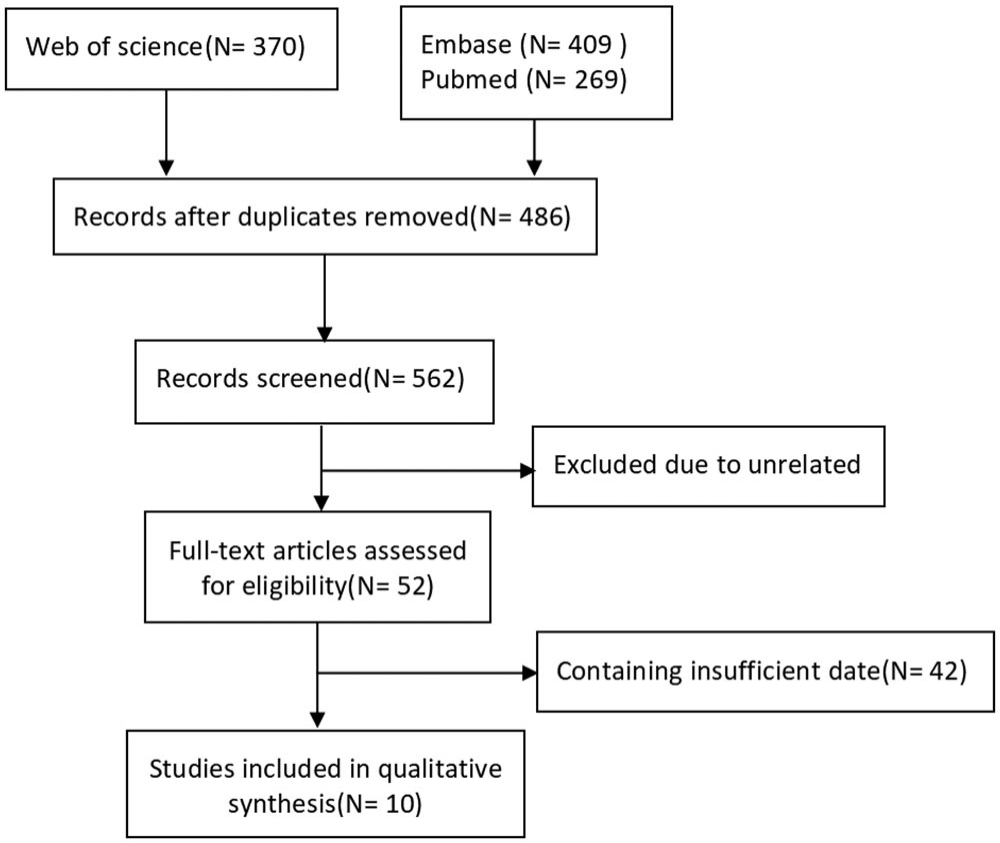
Flowchart of the study selection process.

A total of 1048 potentially relevant studies were obtained from electronic databases and citations of reference lists, out of which 10 cohort studies [12,21–29] from 5 countries met the inclusion criteria. Three of the studies were conducted in China. A detailed illustration of the study enrollment is presented in Figure 1.

### 3.2. Characteristics of enrolled studies

Of the 10 articles included, 8 articles reported all-cause mortality [12,22–25,27–29], and 2 articles reported cardiovascular events [21,22,27] (Table 1). The studies included were scored according to the NOS standard quality index table. The Cohen’s kappa coefficient of agreement during the quality assessment was 0.483 (95% CI 0.18–0.78), indicating moderate agreement.

**Table 1.** Characteristics of included studies.

Study	Country	Design	CKD stage	Number of patients	Participant (male, %)	Age means	Follow-up (month)	Comparison	All-cause mortality (%)	Outcome assessment, HR (95%CI)
Woziwodzka et al. 2019	Poland	Pro	Pre-dialysis and HD	84	59.5	61.5	60	≥3.9, <3.9	38.1	A_1_:2.23(1.10–4.5) A_2_:1.26 (1.06–1.51)
Yaprak et al. 2016	Turkey	Pro	HD	80	40.0	56.8 ± 18.1	24	≥2.52, <2.52	26.2	A_1_:1.54(0.39–6.10)
Sato et al. 2017	Japan	Retro	HD	78	65.4	63.4 ± 11.7	15	≥3.5, <3.5	6.4	A_1_:1.28(1.02–1.60)
An et al. 2012	China	Pro	PD	138	58.7	53 ± 17	38	>3.5, ≤3.5	29	A_1_:1.78(1.38–3.80)
Neuen et al. 2015	Australia	Pro	HD	170	60.0	54 ± 11	37	/	32	A_2_:1.4(1.2–1.6)
Li et al. 2017	China	Pro	HD	268	55.6	48.7 ± 10.9	36	/	32.8	A_2_:1.70(1.29–2.23)
Tatar et al. 2016	Turkey	Pro	Pre-dialysis	165	63.2	73.8 ± 6.1	30 ± 13	/	18.7	A_2_:1.23(1.02–1.47)
Solak et al. 2012	Turkey	Pro	Pre-dialysis	225	47.6	50.2 ± 12.6(NL*R* < 2.81); 48.3 ± 12.2(NL*R* ≥ 2.81)	39	≥2.81, <2.81	8	B:1.5(1.32–1.71)
Abe et al. 2015	Japan	Pro	HD/PD	86	67.4	57.6 ± 11.5	38.7	>3.72, <3.72	10.5	B:2.54(1.09–6.43)
Chen et al. 2016	China	Pro	Pre-dialysis	148	56.1	68.3 ± 10.1	8.6 ± 7.8	≥3.76, <3.76	25.7	A_1_:2.23(1.03–4.82)

Pro: Prospective; Retro: Retrospective; HD: hemodialysis; PD: Peritoneal dialysis; A_1_: all-cause mortality (NLR as a categorical variable); A_2_: all-cause mortality (NLR as a continuous variable); B: cardiovascular events.

### 3.3. Incidence of all-cause mortality

**Figure 2.**
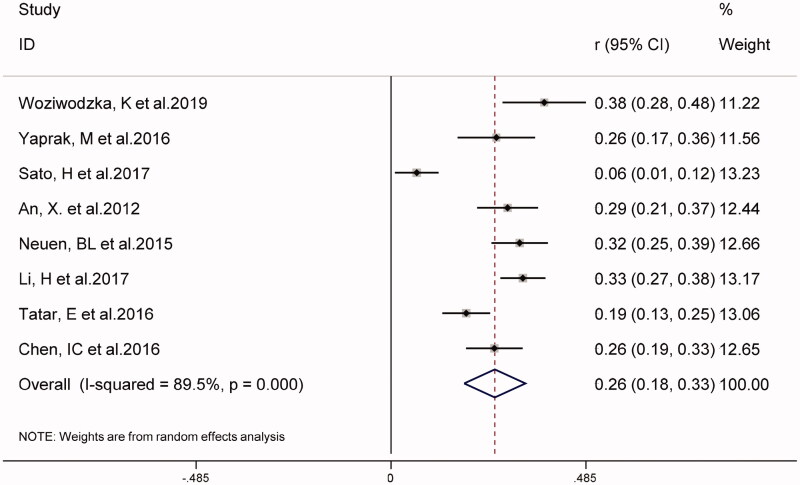
The combined prevalence of all-cause death in CKD patients.

**Figure 3.**
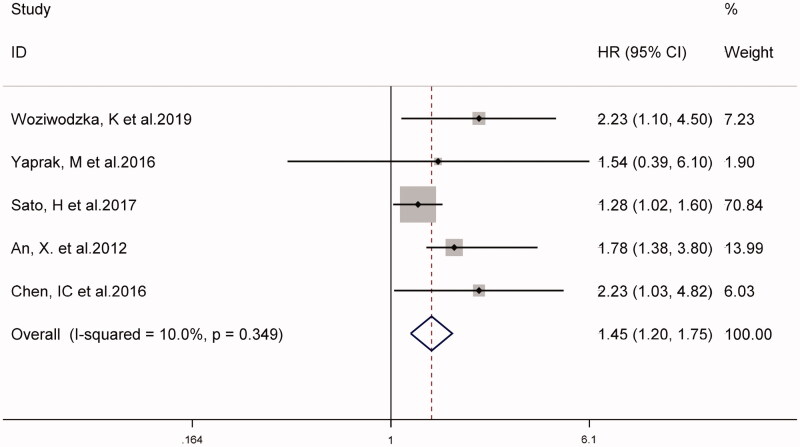
Forest plot for the association between NLR and all-cause mortality in CKD (NLR used as a categorical variable).

In eight studies, the combined prevalence of all-cause mortality in patients with CKD was 26% (95% CI 18–33%), as shown in Figure 2. The overall *I*^2^ was 89.5%, *p* < 0.001. As shown in Figure 3, five studies on NLR as a categorical variable and the risk of all-cause mortality in patients with CKD (weight: 1.90–70.84%), showed that a higher NLR may increase the risk of all-cause mortality in patients with CKD (HR 1.45, 95% CI 1.20–1.75, *I*^2^ = 10.0%). Similarly, in four studies using NLR as a continuous variable (weight: 10.80–39.11%), for every 1-unit increase in NLR, the risk of all-cause mortality in patients with CKD increased by 1.35 times (HR 1.35, 95% CI 1.23–1.48, *I*^2^ = 34.1%), (supplementary Figure S1).

### 3.4. Association between high NLR and cardiovascular events

**Figure 4.**
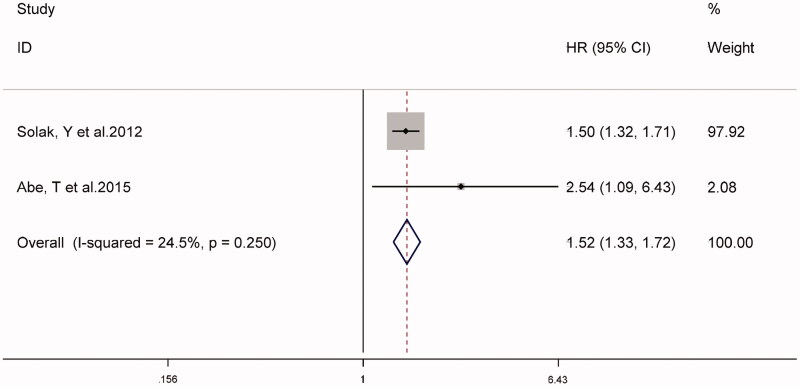
Forest plot for the association between NLR and cardiovascular events.

The combined prevalence of cardiovascular events in patients with CKD in two studies was 36% (95% CI 22–50%, *I*^2^ = 81.3%, *p* < 0.001) (supplementary Figure S2). Analysis of the fixed-effect models shown in Figure 4 suggested that high NLR might be associated with an increased risk of cardiovascular events in patients with CKD (HR 1.52, 95% CI 1.33–1.72, *I*^2^ = 24.5%).

### 3.5. Subgroup analysis

Table 2 shows the detailed results stratified by characteristics of study design, age, country, CKD stage, follow-up, and NLR grouping. In the subgroup analyses, only the study design (prospective and retrospective) groups were statistically significant (*p* = 0.047), which may be the cause of the heterogeneity in our research results.

**Table 2.** Subgroup analysis of the relationship between NLR and all-cause mortality in CKD patients.

Study	No of studies	HR (95% CI)	*I*^2^	*p*^a^	*p*^b^
Design					
Prospective	4	1.95(1.38–2.77)	0%	0.920	0.047
Retrospective	1	1.28(1.02–1.60)	–	–	
Age mean(year)					
<60	2	1.75(1.09–2.81)	0%	0.844	0.396
≥60	3	1.40(1.14–1.72)	45.7%	0.158	
Country					
China	2	1.90(1.25–2.91)	0%	0.632	0.156
Others	3	1.35(1.09–1.67)	9%	0.333	
Male (%)					
<55	1	1.54(0.39–6.07)	–	–	0.932
≥55	4	1.45(1.19–1.75)	32.4%	0.218	
CKD stage					
Pre-dialysis	1	2.23(1.03–4.82)	–	–	0.217
Dialysis	3	1.36(1.11–1.66)	0%	0.498	
Both	1	2.23(1.10–4.51)	–	–	
Follow-up period (months)					
<24	2	1.34(1.08–1.66)	45.4%	0.176	0.133
≥24	3	1.89(1.27–2.80)	0%	0.838	
NLR grouping					
<3.5	2	1.75(1.09–2.81)	0%	0.844	0.396
≥3.5	3	1.40(1.14–1.72)	45.7%	0.158	

a: Heterogeneity within subgroups; b: heterogeneity between two subgroups.

### 3.6. Sensitivity analysis

Sensitivity analysis was based on the NLR as a categorical variable. Further exclusion of any single study showing all-cause mortality did not appear to alter the heterogeneity and combined HR, which suggested that the association between NLR and the increased risk of all-cause mortality in patients with CKD is reliable (supplementary Table S2).

### 3.7. Publication bias

**Figure 5.**
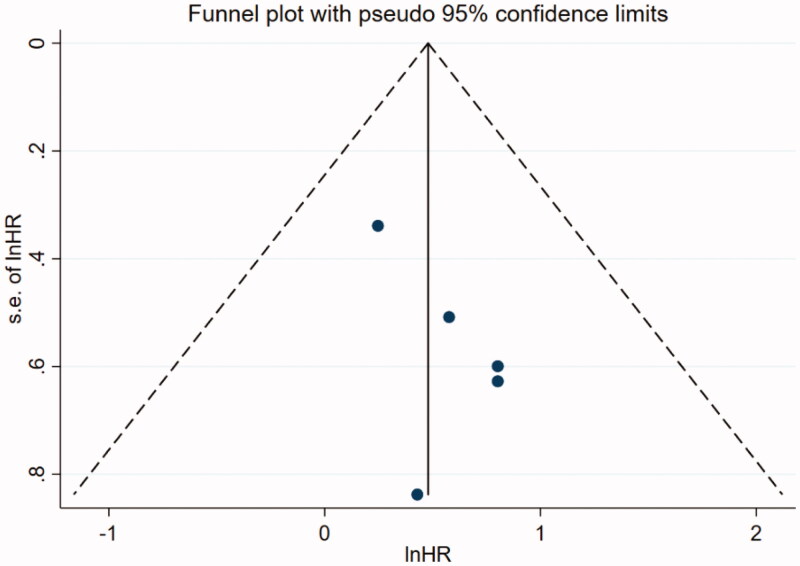
Funnel plot of results of five studies based on the result of NLR and risk of all-cause mortality.

The funnel plot is shown in Figure 5. There was no bias observed in studies on the association of NLR with mortality (Egger’s test *p* = 0.172).

## 4. Discussion

Recently, the relationship between NLR and the prognosis of patients with CKD has gained a lot of attention. Our meta-analysis of 10 cohort studies provided evidence that NLR is a predictor of all-cause mortality and cardiovascular events in patients with CKD. Compared with the lower NLR, the higher NLR had a significantly increased risk of all-cause mortality (HR 1.45, 95% CI 1.20–1.75) and cardiovascular events (HR 1.52, 95% CI 1.33–1.72). Similar results were obtained when NLR was used as a continuous variable (HR 1.35; 95% CI 1.23-1.48).

Our subgroup analysis revealed whether CKD or dialysis patients would show an increased risk of all-cause mortality and cardiovascular events affected by the NLR. Moreover, the heterogeneity between groups was small. However, NLR has been shown to independently predict the risk of ESRD in patients with advanced CKD, and to a small extent, in patients with early CKD [11].

Because of the low heterogeneity observed in the study, a subgroup analysis was performed to detect potential sources of heterogeneity. The results of subgroup analysis showed that NLR was related to the high risk of death in patients with CKD in different types of studies, and that prospective studies may contribute to heterogeneity. This means that prospective cohort studies provide a higher level of evidence than retrospective cohort studies. Well-designed prospective cohort studies with larger samples need to be conducted, to evaluate the relationship between NLR and the prognosis of patients with CKD, or well-designed randomized controlled trials to examine whether NLR intervention can improve the prognosis of patients with CKD should be performed.

Neutrophils are the most abundant immune cells in the human body and are the first line of defense against infection and tissue damage. They can kill pathogens by chemotaxis, phagocytosis, and direct sterilization [30]. In recent years, neutrophil extracellular trap (NET), a new neutrophil antibacterial method, has been discovered, which can not only capture and kill pathogens by releasing nucleic acid substances and granulocyte proteins into a network structure but also is related to multiple pathophysiological processes of human body, such as inflammation, tumor cell migration, ischemia/reperfusion injury, autoimmunity [31–33], etc. It is noteworthy that neutrophils mediate the inflammatory response in kidney injury by various biochemical mechanisms, leading to further tissue damage [34]. These mechanisms include the release of reactive oxygen species, myeloperoxidase, and proteolytic enzymes which can affect kidney function [35]. Studies have reported that anti-inflammatory treatment can protect the kidneys [36,37].

The lymphocyte is a kind of cell line with immune recognition function, mainly exists in the circulating lymph fluid in lymphatic vessels, and is an important cell component of the immune response function of the body. As the main enforcer of almost all the immune functions of the lymphatic system, lymphocytes are the frontline soldiers to fight against external infections and monitor the variation of cells in the body. Under stress, the release of cortisol and catecholamines in blood increases, which leads to bone marrow suppression, and thus the proliferation and differentiation of lymphocytes are reverentially regulated and the apoptosis of lymphocytes is aggravated. In a state of severe inflammation, hypo-lymphocytosis may even occur. Inflammatory response leads to lymphocyte apoptosis, lymphocyte differentiation, and down-regulated proliferation, and neurohumoral activation leads to a decreased immune regulation. In addition, lymphocyte counts have been used as indicators of nutritional status, and poor nutritional status is a risk factor for all-cause mortality [3,38].

Inflammation is commonly observed in patients with CKD [39]. It is well known that vascular endothelium is more likely to form atherosclerotic plaques and calcification foci when in an inflammatory state, which in turn induces cardiovascular events and even death in patients with chronic kidney disease [40]. Inflammation is commonly observed in patients with CKD [39]. NLR affects two immune pathways involving the increase and decrease in counts of neutrophils and lymphocytes, resulting in an inflammatory imbalance that ultimately leads to death in patients with CKD. The combination of elevated neutrophil counts and low levels of lymphocytes, detected as a single comprehensive marker of inflammation can provide more information. Previous studies mentioned that treatment to reduce vascular calcification patients with renal disease may offset the excessive risk of cardiovascular disease [41,42].

NLR outperformed other white blood cells in predicting both short-and long-term mortality. The number of white blood cell or lymphocyte subtypes can be altered by various physiological, pathological, and physical factors, but the NLR may remain stable. Since certain traditional inflammatory cytokines, such as high sensitivity CRP, interleukin 6, and tumor necrosis factor alpha, are limited by unconventional detection, NLR will be more widely used in clinical practice.

Our study had a few limitations that must be underlined. First, due to the limited number of articles that meet the criteria, this research is based on a small number of studies. Second, all included studies include prospective and retrospective cohort studies. Lack of information and data are inevitable. Therefore, we did not use the Grading of Recommendations Assessment, Development and Evaluation (GRADE) system to score the quality of each outcome indicator. Finally, there are differences between the enrolled researches, such as NLR grouping, follow-up times and blood sampling time. Reliable conclusions still need to be confirmed by studies with large sample sizes, long follow-up times, and multiple centers and regions.

## 5. Conclusion

Our meta-analysis indicates that high NLR in patients with CKD increases the risk of all-cause mortality and cardiovascular events during follow-up. These results suggest that NLR is a reliable inflammation mediator to predict poor prognosis in patients with CKD. More sophisticated trials are needed to determine whether neutrophil or lymphocyte counts are appropriate therapeutic targets to improve the clinical outcome of patients with CKD.

## Supplementary Material

Supplemental Material
