# Supplementary material for: Neutrophil-to-lymphocyte ratio in relation to the risk of all-cause mortality and cardiovascular events in patients with chronic kidney disease: a systematic review and meta-analysis
Source: Ren Fail. 2020 Oct 20;42(1):1059–66. doi: 10.1080/0886022X.2020.1832521 (PMC7668415; doi:10.1080/0886022X.2020.1832521)
Supplement: Supplemental Material [file IRNF_A_1832521_SM1210.pdf]

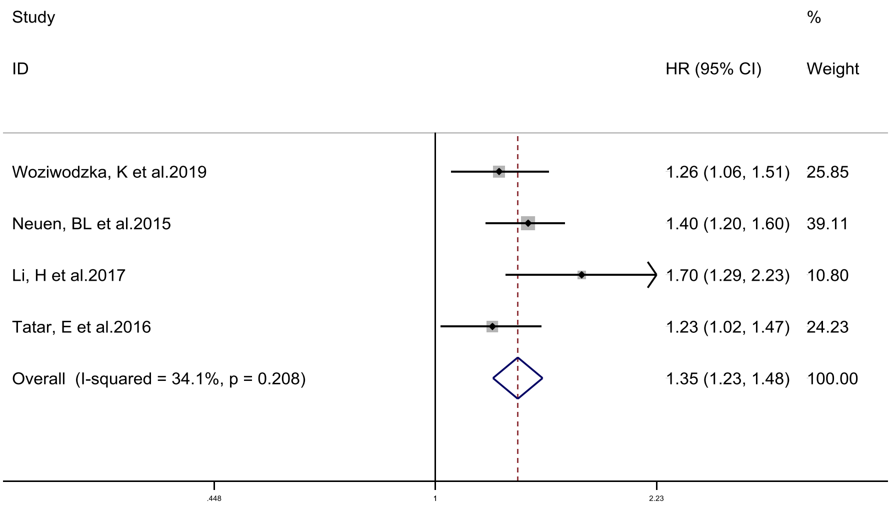

Figure S1. Forest plot for the association between NLR and all-cause mortality in patients with CKD (NLR used as a continuous variable).

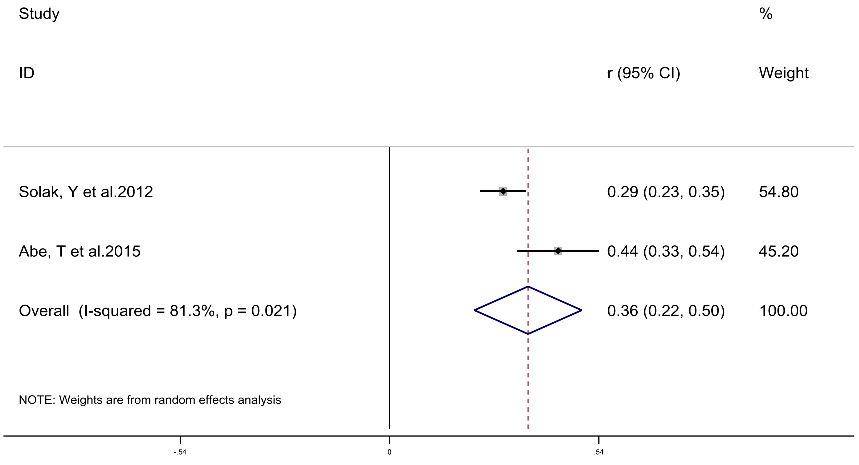

Figure S2. The combined prevalence of cardiovascular events in patients with CKD.

Table S1. Two researchers based on the initial scores of the Newcastle-Ottawa Scale (NOS) Standard Quality Index Table.

Study	Wen-Man Zhao	Shu-Man Tao
Woziwodzka, K et al.2019	9	8
Yaprak, M et al.2016	9	8
Sato, H et al.2017	8	7
An, X. et al.2012	6	6
Neuen, BL et al.2015	9	8
Li, H et al.2017	8	7
Tatar, E et al.2016	8	7
Solak, Y et al.2012	6	6
Abe, T et al.2015	8	7
Chen, IC et al.2016	7	7

Table S2. Sensitivity analysis of the relationship between NLR and all-cause mortality in CKD

Study	HR	95% CI
Woziwodzka, K et al.2019	1.40	1.15-1.70
Yaprak, M et al.2016	1.45	1.19-1.75
Sato, H et al.2017	1.95	1.38-2.77
An, X. et al.2012	1.4	1.14-1.72
Chen, IC et al.2016	1.41	1.16-1.71
	1.45	1.20-1.75
